# Ascorbic Acid Chemosensitizes Colorectal Cancer Cells and Synergistically Inhibits Tumor Growth

**DOI:** 10.3389/fphys.2018.00911

**Published:** 2018-07-23

**Authors:** Ana S. Pires, Cláudia R. Marques, João C. Encarnação, Ana M. Abrantes, Inês A. Marques, Mafalda Laranjo, Rui Oliveira, João E. Casalta-Lopes, Ana C. Gonçalves, Ana B. Sarmento-Ribeiro, Maria F. Botelho

**Affiliations:** ^1^Biophysics Institute, CNC.IBILI, Faculty of Medicine, University of Coimbra, Coimbra, Portugal; ^2^Faculty of Sciences and Technology, University of Coimbra, Coimbra, Portugal; ^3^Institute for Clinical and Biomedical Research Area of Environment Genetics and Oncobiology, Faculty of Medicine, University of Coimbra, Coimbra, Portugal; ^4^Department of Pathology, Centro Hospitalar e Universitário de Coimbra, Coimbra, Portugal; ^5^Oncobiology and Hematology Laboratory, Applied Molecular Biology and University Clinic of Hematology, Faculty of Medicine, University of Coimbra, Coimbra, Portugal; ^6^Department of Hematology, Centro Hospitalar e Universitário de Coimbra, Coimbra, Portugal

**Keywords:** vitamin C, ascorbic acid, colorectal cancer, chemosensitizing effect, synergy, oxaliplatin

## Abstract

Colorectal cancer (CRC) is continuously classified as one of the most incidental and mortal types of cancer worldwide. The positive outcomes of the conventional chemotherapy are frequently associated with high toxicity, which often leads to the suspension of the treatment. Growing evidences consider the use of pharmacological concentrations of ascorbic acid (AA), better known as vitamin C, in the treatment of cancer. The use of AA in a clinical context is essentially related to the adoption of new therapeutic strategies based on combination regimens, where AA plays a chemosensitizing role. The reduced sensitivity of some tumors to chemotherapy and the highly associated adverse effects continue to be some of the major obstacles in the effective treatment of CRC. So, this paper aimed to study the potential of a new therapeutic approach against this neoplasia with diminished side effects for the patient. This approach was based on the study of the combination of high concentrations of AA with reduced concentrations of drugs conventionally used in CRC patients and eligible for first and second line chemotherapeutic regimens, namely 5-fluorouracilo (5-FU), oxaliplatin (Oxa) or irinotecan (Iri). The evaluation of the potential synergy between the compounds was first assessed *in vitro* in three CRC cell lines with different genetic background and later *in vivo* using one xenograft animal model of CRC. AA and 5-FU act synergistically *in vitro* just for longer incubation times, however, *in vivo* showed no benefit compared to 5-FU alone. In contrast to the lack of synergy seen in *in vitro* studies with the combination of AA with irinotecan, the animal model revealed the therapeutic potential of this combination. AA also potentiated the effect of Oxa, since a synergistic effect was demonstrated, in almost all conditions and in the three cell lines. Moreover, this combined therapy (CT) caused a stagnation of the tumor growth rate, being the most promising tested combination. Pharmacological concentrations of AA increased the efficacy of Iri and Oxa against CRC, with promising results in cell lines with more aggressive phenotypes, namely, tumors with mutant or null P53 expression and tumors resistant to chemotherapy.

## Introduction

Colorectal cancer (CRC) is continuously classified as one of the most incidental and mortal types of cancer worldwide (Siegel et al., 2014; Ahn et al., 2016). First and second line therapeutic options for CRC are based on drug combinations that include fluoropyrimidines, oxaliplatin (Oxa) and irinotecan (Iri) (Oostendorp et al., 2010; Labianca et al., 2013).

5-Fluorouracil (5-FU) is a conventional chemotherapeutic drug extensively used in the treatment of CRC, since its introduction in the 1960s (Boland et al., 2000). By inhibiting thymidylate synthase activity, it mainly affects DNA and also incorporates into RNA (Kuilenburg and Van, 2004). Oxa, a platinum-based drug, has the capacity of forming cross-linking adducts preventing DNA replication and transcription, while Iri exerts its cytotoxic effects through the inhibition of topoisomerase I, resulting in double-stranded DNA breaks and cell death (Guichard et al., 1999; Raymond et al., 2002). Besides the positive outcomes of the use of these drugs, they go hand in hand with toxicity, which often leads to the suspension of the treatment.

Growing evidences consider the use of natural compounds in the prevention and treatment of cancer. The intravenous application of high concentrations of vitamin C (ascorbic acid, AA), better known as vitamin C, has been used since the 1970s for cancer treatment (Venturelli et al., 2015). Its therapeutic potential has been supported by a large and consistent body of evidences from *in vitro* (Riordan et al., 1995; Chen et al., 2005; Klingelhoeffer et al., 2012; Kawada et al., 2013; Mastrangelo, 2013; Mastrangelo et al., 2013, 2015; Fukui et al., 2015; Pires et al., 2016), *in vivo* (Chen et al., 2007; Verrax and Calderon, 2009; Yeom et al., 2009; Espey et al., 2011; Serrano et al., 2015) and clinical studies (Ohno et al., 2009; Monti et al., 2012; Welsh et al., 2013; Ma et al., 2014; Hoffer et al., 2015).

Ascorbic acid might act as a way to deliver hydrogen peroxide (H_2_O_2_) to the tissues, ultimately leading to tumor cell death via different pathways (Chen et al., 2007; Du et al., 2010). We have previously reported that pharmacological concentrations of AA were capable of inducing anti-proliferative, cytotoxic and genotoxic effects on three colon cancer cell lines, being the mechanism of action dependent on the cell type (Pires et al., 2016).

The use of AA in a clinical context is essentially related to the adoption of new therapeutic strategies based on combination regimens where AA plays a chemosensitizing role. While some studies determine that it might protect cancer cells from chemotherapy, others acknowledge that pharmacological concentrations of AA can sensitize cancer cells to chemotherapy, enhancing its antineoplastic effect. Its synergistic effect with conventional chemotherapeutic drugs is a fact already reported, in various types of cancer, by numerous authors, namely in pancreatic (Espey et al., 2011), prostate (Gilloteaux et al., 2014), lung (Lee et al., 2017), breast (Kurbacher et al., 1996; Wu et al., 2017) and ovarian (Ma et al., 2014) cancers.

Given the advantages of using combined regimens to treat cancer, we aim to evaluate *in vitro* and *in vivo* the therapeutic potential of the combination of AA with 5-FU, Oxa or Iri in three CRC cell lines and in one xenograft animal model of CRC.

## Materials and Methods

### Cell Culture

Two human cell lines of CRC, C2BBe1 [clone of Caco-2] (ATCC Cat# CRL-2102, RRID:CVCL_1096) and WiDr (ATCC Cat# CCL-218, RRID:CVCL_2760), were cultured in Dulbecco’s Modified Eagle’s Medium, DMEM (Sigma) and the another one, LS1034 (ATCC Cat# CRL-2158, RRID:CVCL_1382), was cultured in Roswell Park Memorial Institute Medium, RPMI-1640 (Sigma). Cells were grown at 37°C with 95% air and 5% CO_2_.

### Cells Treatment

To evaluate the effect of AA combined with one of the three chemotherapeutic agents, namely 5-FU, Oxa and Iri, and ascertain the existence of synergy, the model proposed by Straetemans et al. (2005) was followed. This ray design model was used to mix the two compounds, in a solution Z, based on a specific equation [Z = *f*A + (1 – *f*) B]. The values of A and B correspond to the IC_50_ (half maximal inhibitory concentration) values of AA and the chemotherapeutic agent, respectively, determined for an incubation period of 96 h. The IC_50_ values for each drug were calculated after cell treatment with rising concentrations of 5-FU (0.5–480 μM), Oxa (1–120 μM) and Iri (5–200 μM). For the assays with 5-FU and Iri, a second control consisting in the solvent for both compounds (DMSO) was considered. For the mixture factor (*f*), which corresponds to the proportion of each compound, the values 0.5 and 0.75 were established.

Seven variations of Z corresponding to different combinations for each cell line were settled, by adopting distinct values of *a* (**Table 1**). The *a* values were determined taking into account the IC_50_ of AA for each cell line, the solubility of AA in water and the acquisition of dose-response curves capable of expressing the existence of synergy. Cells were incubated with the seven variations of Z for *f* = 0.5 and *f* = 0.75 during 24, 48, 72, and 96 h, being cell proliferation evaluated afterward. The experimental design proposed by Straetemans et al. (2005) also allowed to determine the combination index (CI) and infer whether the CT was synergistic (CI < 1), additive (CI = 1) or antagonistic (CI > 1).

**Table 1:** Concentrations of ascorbic acid (AA), 5-Fluorouracil (5-FU), oxaliplatin (Oxa), and irinotecan (Iri) present in each mixture, for *f* = 0.75, to evaluate cell proliferation of C2BBe1, LS1034, and WiDr cell lines.

			AA + 5-FU		AA + Oxa		AA + Iri	
Cell line	Conditions	Variations of Z (*a*Z)	[AA] (mM)	[5-FU] (μM)	[AA] (mM)	[Iri] (μM)	[AA] (mM)	[Oxa] (μM)
C2BBe1	B1	0.25Z	0.15	1.26	0.15	0.14	0.15	0.74
	B2	0.5Z	0.31	2.51	0.31	0.28	0.31	1.48
	B3	Z	0.61	5.03	0.61	0.55	0.61	2.97
	B4	2Z	1.23	10.05	1.23	1.11	1.23	5.93
	B5	4Z	2.45	20.10	2.45	2.21	2.45	11.87
	B6	6Z	–	–	3.68	3.32	3.68	17.80
	B7	8Z	4.91	40.20	4.91	4.42	4.91	23.73
LS1034	B1	0.25Z	1.01	0.42	1.01	0.13	1.01	0.30
	B2	0.5Z	2.01	0.84	2.01	0.25	2.01	0.59
	B3	Z	4.03	1.67	4.03	0.50	4.03	1.19
	B4	1.5Z	6.04	2.51	6.04	0.75	6.04	1.78
	B5	2Z	8.05	3.35	8.05	1.01	8.05	2.38
	B6	3Z	12.08	5.02	12.08	1.51	12.08	3.57
	B7	4Z	–	–	16.10	2.01	16.10	4.76
WiDr	B1	0.25Z	1.11	0.18	1.11	0.51	1.11	0.11
	B2	0.5Z	2.21	0.35	2.21	1.03	2.21	0.22
	B3	Z	4.43	0.71	4.43	2.05	4.43	0.44
	B4	1.5Z	6.64	1.06	6.64	3.08	6.64	0.66
	B5	2Z	8.86	1.41	8.86	4.11	8.86	0.88
	B6	3Z	13.28	2.12	13.28	6.16	13.28	1.33
	B7	4Z	–	–	17.71	8.21	17.71	1.77

*‘a’ Represents the increasing values multiplied by the constant Z to obtain the different combinations of the ray design.*

The effect of CT on cell viability, types of induced cell death and cell cycle deviations was determined using three different combinations (**Table 2**) and an incubation period of 48 h. These most promising combinations were also used to evaluate mitochondrial membrane potential (Δψ_m_) and the expression of BAX, BCL-2, P53 and caspase-9.

**Table 2:** Concentrations of AA, 5-FU, Oxa, and Iri used in combined therapy (CT) for C2BBe1, LS1034 and WiDr cell lines for flow cytometry and western blot studies.

	AA + 5-FU			AA + Oxa			AA + Iri		
Cell line	Conditions	[AA] (mM)	[5-FU] (μM)	Conditions	[AA] (mM)	[Oxa] (μM)	Conditions	[AA] (mM)	[Iri] (μM)
C2BBe1	B4	1.23	10.05	B3	0.61	0.55	B4	1.23	5.93
LS1034	B5	8.05	3.35	B3	4.03	0.50	B3	4.03	1.19
WiDr	B5	8.86	1.41	B3	4.43	2.05	B4	6.64	0.66

### SRB Assay

The effect of the seven combinations on cell proliferation was determined through the colorimetric sulphorhodamine B (SRB) assay, a negatively charged pink aminoxanthine dye that gives a measure of protein synthesis (Houghton et al., 2007). The protocol was performed as previously described by Pires et al. (2016). The IC_50_ values were determined through sigmoid fitting.

### Flow Cytometry

Changes in cell cycle, cell viability and cell death, Δψ_m_ and BAX, BCL-2 expression were determined by flow cytometry. At least 10^4^ events were collected using Cell Quest software (Becton Dickinson, San Jose, CA, United States) and analyzed using Paint-a-Gate software (Becton Dickinson, San Jose, CA, United States).

Cell cycle was evaluated using propidium iodide (PI), which has the capacity to intercalate DNA enabling its quantification (Mansilla et al., 2003). The protocol was performed as described by Brito et al. (2016). Results are expressed as the percentage of cells at each phase of cell cycle (G0/G1, S and G2/M phases) estimated from their histograms of DNA content. The apoptotic peak (pre-G1) was also quantified.

Cell viability and types of cell death were assessed through annexin-V/propidium iodide (AV/PI) incorporation assay, as described by Tavares-da-Silva et al. (2016). The results are expressed as the percentage of viable, early apoptotic, late apoptotic/necrotic and necrotic cells.

The lipophilic cationic molecule 5,5′,6,6′-tetrachloro-1,1′,3,3′-tetraethyl benzimidazolocarbocyanine iodide (JC-1, Sigma) was used to assess mitochondrial membrane potential (Δψ_m_), according to a previously published protocol (Mendes et al., 2016). Results are represented as aggregates(A)/monomers(M) fluorescence intensities ratio relative to control.

For the evaluation of BAX and BCL-2 expression levels, 10^6^ cells were fixed and permeabilized, followed by labeling with the monoclonal antibodies anti-Bax-PE (Santa Cruz Biotechnology, Cat# sc-20067 PE) and anti-Bcl2-FITC (Santa Cruz Biotechnology, Cat# sc-509 FITC). The results are expressed as BAX/BCL-2 ratio of fluorescence intensities relative to control.

### Western Blot Analysis

Western blot enabled the determination of the effect of the combination of AA with each chemotherapeutic drug on the expression of caspase-9 and P53. Total protein extracts were prepared using RIPA buffer supplemented with complete^TM^ Mini (Roche) and protein concentration was determined by BCA^TM^ protein assay (Pierce). Western blot analyses were done according to a previously described protocol (Pires et al., 2016). The primary antibodies anti-caspase-9 (Santa Cruz Biotechnology Cat# sc-8355, RRID:AB_2071323), anti-P53 (Santa Cruz Biotechnology Cat# sc-47698, RRID:AB_628083) and anti-actin (Sigma, A5141) and the secondary antibodies goat-anti-mouse (GE Healthcare) and goat anti-rabbit (Santa Cruz Biotechnology Cat# sc-2007, RRID:AB_631740) were used. Standard protein markers (Nzytech, MB09002) were also used. Membranes were scanned using Typhoon FLA 9000 equipment.

### *In Vivo* Studies

To understand if the combination of AA with each chemotherapeutic drug was effective to reduce tumor volume, heterotopic xenografts were developed by inoculating cell suspensions of 7 × 10^6^ WiDr cells on the back of *Balb/c nu/nu* mice (Charles River Laboratories International, Inc.). The body weight of all animals, tumor volume, as well as animal behavior and general health were monitored during several days. When tumors reached a volume of 300–500 mm^3^, treatments were initiated being day 0 considered the first day of the therapy. Eight animal groups were defined: (I) control group, which was not subjected to any treatment; (II) AA group, subjected to treatment with an aqueous solution of AA (150 mg/kg), according to Mamede et al. (2012); (III) 5-FU group, subjected to therapy with an aqueous solution of 5-FU (2 mg/kg), according to Pegram et al. (1999); (IV) AA + 5-FU group, treated with the combination of AA and 5-FU; (V) Oxa group, treated with an aqueous solution of Oxa (4 mg/kg), in agreement with the protocol of Al Moundhri et al. (2013); (VI) AA + Oxa group, subjected to administration of AA and Oxa in combination; (VII) Iri group, exposed to the administration of a solution of Iri in 0.9% NaCl (100 mg/kg), homologous to the methodology of Guichard et al. (1998); (VIII) AA + Iri group, CT of AA and Iri. The association of the protocols for administration of AA and each chemotherapeutic drug led to the methodology adopted for the CT. After therapy, mice were sacrificed by cervical dislocation. During the experiments, animals were kept under sterile conditions in filtertop cages (cycles of 12 h of dark/light), under constant temperature and humidity, with access to a sterilized diet and water *ad libitum*. This study was carried out in accordance with the guidelines of the Portuguese Society of Animal Science Laboratory (SPCAL) and approved by the Ethics Committee of the Faculty of Medicine of University of Coimbra.

### Histological Analysis

Tumors retrieved from mice were fixed in 10% formalin (VWR Chemicals, 90240) and analyzed. Samples were then embedded in paraffin, cut into 4 μm sections. Histological examination was performed using a light microscope – Nikon Eclipse 50i, and images obtained using a Nikon-Digital Sight DS-Fi1 camera. Immunohistochemistry studies were performed on a representative block of the lesion, performed on Ventana Marker Platform Bench Mark ULTRA IHC/ISH, with an indirect multimeric detection system, biotin free, peroxidase conjugated, with anti-KI-67 antibody (Santa Cruz Biotechnology Cat# sc-23900, RRID:AB_627859).

The Ki-67 proliferative index was counted accordingly to the manual counting of camera-captured/printed image method defined for neuroendocrine tumors (Reid et al., 2015): each slide was manually scanned under a × 10 objective, for selection of the area with greatest Ki67 positivity (hot spot), with posterior photograph and print. The color image was printed on plain white paper and Ki67-negative and -positive tumor cells were then visualized and immediately marked/crossed off once counted. Light brown/pale staining nuclei were ignored for the purpose of counting.

### Statistical Analysis

The statistical analyses were performed using software IBM^TM^ SPSS^TM^ 22.0 (SPSS, RRID:SCR_002865). The evaluation of the normality of the quantitative variables distribution and variances homogeneity was done according to Shapiro-Wilk and Levene tests, respectively. Parametric tests were used when in the presence of a normal distribution and variance homogeneity and non-parametric tests otherwise. Comparison of quantitative variables between two groups was performed using parametric Student *t*-test and, in more than two groups, was obtained using one-factor ANOVA test. *Post hoc* analyses were made using the Games-Howel test (with variances homogeneity) and otherwise using the Bonferroni correction. For all comparisons, a significance of 0.05 was considered.

## Results

### AA and Conventional Drugs Act Synergistically

The combination model allowed the determination of the IC_50_ values of the drugs when present in combination with AA. These values are compared with the IC_50_ values of the respective drugs obtained in monotherapy, i.e., in the absence of AA (**Table 3**). This model also allowed the determination of the CI (**Table 4**).

**Table 3:** IC_50_ values (in μM) of 5-FU, Oxa, and Iri, obtained in monotherapy (MT) and in combined therapy with AA (CT), with *f* = 0.5 or *f* = 0.75, obtained after treatment of C2BBe1, LS1034, and WiDr cells for 24, 48, 72, and 96 h.

		5-FU			Oxa			Iri		
Cell line	Time (h)	MT	CT (*f* = 0.5)	CT (*f* = 0.75)	MT	CT (*f* = 0.5)	CT (*f* = 0.75)	MT	CT (*f* = 0.5)	CT (*f* = 0.75)
C2BB e1	24	^(a)^	^(a)^	^(a)^	115.2	0.7	0.4	^(b)^	8.8	3.1
	48	^(b)^	18.1	6.9	5.2	1.0	0.3	132.6	17.0	5.0
	72	58.1	10.7	5.2	3.1	0.4	0.1	75.9	10.3	2.9
	96	20.1	3.1	1.3	2.2	0.5	0.2	11.9	6.1	2.2
LS1034	24	^(a)^	^(a)^	^(a)^	31.9	3.9^(c)^	0.5	83.4	3.2	^(b)^
	48	238.0	6.3	2.6	5.9	0.5	0.4	47.5	1.4^(c)^	0.5
	72	15.4	6.3	1.4	1.9	1.2	0.3	8.1	4.3	1.1^(c)^
	96	6.7	1.5^(c)^	0.8	2.0	0.6	0.3	4.8	1.5^(c)^	0.5
WiDr	24	^(a)^	^(a)^	^(a)^	^(b)^	7.7	4.9	^(b)^	^(b)^	1.4
	48	442.4	3.4	1.4	21.0	2.6	1.5	38.7	1.6	0.8
	72	10.5	1.6	1.1	10.9	0.8	0.2	13.9	1.8	^(b)^
	96	2.8	0.9	0.5	8.2	0.8	0.4	1.8	0.9^(c)^	0.4

*^(a)^ Represents non-evaluated conditions, ^(b)^ represents conditions for which it was not possible to determine the IC_50_ value, and ^(c)^ represents conditions for which the obtained r^2^ was lower than 0.9.*

**Table 4:** Combination index (CI) values obtained for C2BBe1, LS1034 and WiDr cells, for *f* = 0.5 and *f* = 0.75, and for each incubation time.

		AA + 5-FU		AA + Oxa		AA + Iri	
Cell line	Time (h)	*f* = 0.5	*f* = 0.75	*f* = 0.5	*f* = 0.75	*f* = 0.5	*f* = 0.75
C2BBe1	24	^(a)^	^(a)^	**0.62**	**0.67**	1.48	1.03
	48	1.80	1.37	**0.93**	**0.47**	2.87	1.68
	72	1.06	1.04	**0.38**	**0.22**	1.74	0.98
	96	**0.31**	**0.26**	**0.46**	**0.37**	1.02	**0.75**
LS1034	24	^(a)^	^(a)^	3.91	1.03	1.35	^(b)^
	48	1.88	1.53	**0.51**	**0.79**	**0.59**	**0.39**
	72	1.88	**0.81**	1.21	**0.61**	1.82	**0.91**
	96	**0.44**	**0.46**	**0.58**	**0.57**	**0.64**	**0.46**
WiDr	24	^(a)^	^(a)^	1.87	2.39	^(b)^	3.23
	48	2.43	1.99	**0.63**	**0.74**	1.80	1.79
	72	1.11	1.61	**0.20**	**0.08**	2.04	^(b)^
	96	**0.61**	**0.71**	**0.20**	**0.17**	1.07	**0.94**

*The values in bold type represent the conditions in which synergistic effect was obtained (CI < 1).*

In general, the presence of AA induced a decrease in the IC_50_ of all drugs in all the studied conditions, compared to the IC_50_ of the same drugs in monotherapy. In addition, the greater the proportion at which AA is present (*f* = 0.75), the lower the IC_50_ of the chemotherapeutic drugs. These facts were observed in the three CRC cell lines.

Despite the inexistence of a synergistic effect for all conditions, 5-FU and Iri proved to act synergistically with AA for greater incubation periods (96 h) and higher AA concentrations (*f* = 0.75). In a general way, the combination of Oxa with AA appears to be the most promising, considering that CI was lower than 1, for all cell lines and for incubation times greater than 48 h. It is also important to note that in LS1034 cells, a cell line described as chemoresistant, AA improved the effect of Oxa and Iri regarding the inhibition of cancer cells proliferation, showing a synergistic effect at 48 h.

### Combined Therapies Induce Alterations in Cell Viability and Cell Cycle

**FIGURE 1:**
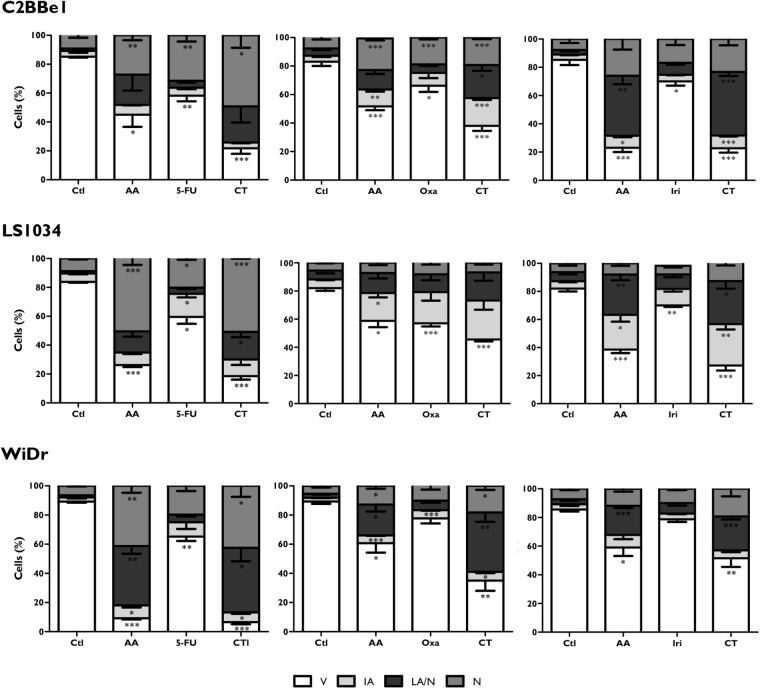
Analysis of cell viability and types of cell death induced in C2BBe1, LS1034, and WiDr cells after exposure to AA and 5-FU, oxaliplatin (Oxa) or irinotecan (Iri) alone or in combined therapy (CT) for 48 h. The results are represented in percentage (%) of viable cells (V), initial apoptosis (IA), late apoptosis/necrosis (LA/N) and necrosis (N). The results express the mean and standard error of at least three independent experiments, in duplicate. Statistically significant differences relative to control are marked with ^∗^*p* < 0.05, ^∗∗^*p* < 0.01 and ^∗∗∗^*p* < 0.001.

Colorectal cancer cell lines were incubated during 48 h with the most promising conditions previously determined (**Table 2**). Results of cell viability and death are shown in **Figure 1**. Relatively to the CT of AA + 5-FU, C2BBe1, LS1034, and WiDr cells were treated, respectively, with B4, B5 and B5 combination for each cell line, according to **Table 2**. Generally, after CT, all cell lines showed a significant decrease in cell viability followed by an increase in cell death, wherein C2BBe1 cells are mostly in late apoptosis/necrosis, LS1034 cells in necrosis and WiDr in both. CT showed to decrease significantly (*p* < 0.001) the viability of C2BBe1, LS1034 and WiDr cells to 21.8, 18.5, and 6.5%, respectively, compared with 5-FU treatment. However, CT did not enhance cell death effects in comparison with AA alone.

In regard to CT of AA + Oxa, the B3 condition of each cell line was used for the treatment, according to **Table 2**. CT of AA + Oxa induced a significant decrease of viability to 38, 45.5, and 27.3%, for C2BBe1, LS1034 and WiDr cells, respectively. CT showed a significant reduction in cell viability, when compared to monotherapies, namely with AA (*p* < 0.001) and Oxa (*p* = 0.003) in C2BBe1 cells, as well as with Oxa in LS1034 cells (*p* = 0.009) and in WiDr cells (*p* < 0.001). This decrease is followed by a significant increment in cell death for all cell lines. However, only C2BBe1 cells showed a statistical decrease in cell death, compared with AA (51.7 ± 2.7%; *p* = 0.048) and Oxa (66.2 ± 4.3%; *p* = 0.003) in monotherapy. This cell death was essentially due to late apoptosis/necrosis, statistically higher than cells treated only with Oxa (*p* = 0.024).

Relatively to CT of AA + Iri, the B4 combination was used for the treatment of C2BBe1 and WiDr cells and B3 for LS1034 cells, according to **Table 2**. After 48 h, CT leads to a significant decrease in cell viability to 22% (*p* < 0.001), 27.2% (*p* < 0.001) and 51.5% (*p* = 0.021) for C2BBe1, LS1034 and WiDr cells, respectively. All cell lines showed an increase in cell death followed by a significant higher number of cells in late apoptosis/necrosis, in comparison with to Iri in monotherapy. There was an increment of cells in early apoptosis for C2BBe1 and LS1034 and in necrosis for LS1034, when compared to the use of Iri alone. CT did not show significant differences compared with AA monotherapy.

**FIGURE 2:**
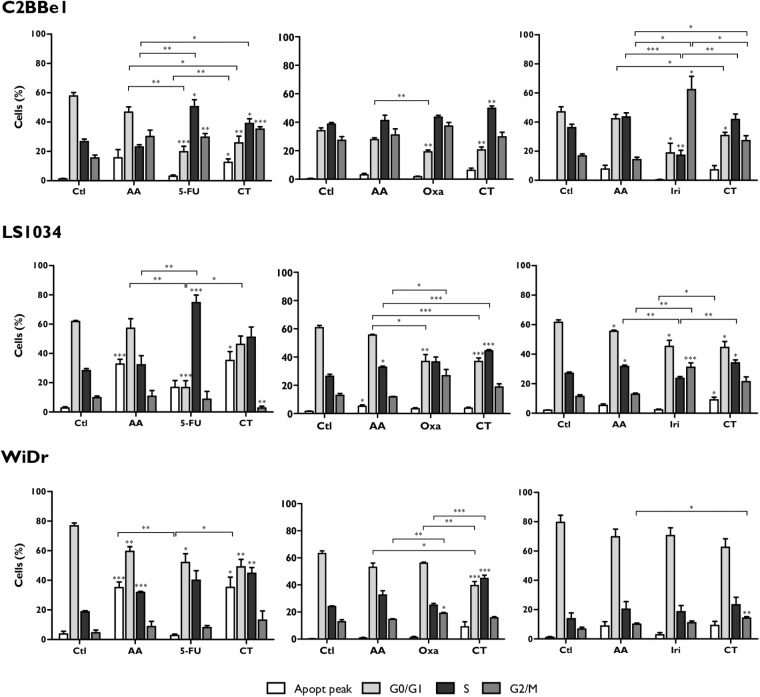
Analysis of cell cycle alterations in C2BBe1, LS1034 and WiDr cells after exposure to AA and 5-FU, Oxa or Iri alone or in CT for 48 h. The results are represented in percentage (%) of cells in the pre-G0/G1 peak and in the G0/G1, S and G2/M phases of the cell cycle. The results express the mean and standard error of at least three independent experiments, in duplicate. Statistically significant differences are marked with ^∗^*p* < 0.05, ^∗∗^*p* < 0.01, and ^∗∗∗^*p* < 0.001.

In regard to cell cycle, as can be seen in **Figure 2**, the combination of AA + 5-FU induces cell cycle arrest in S-phase in C2BBe1 cells (*p* < 0.05), followed by a decrease in the percentage of cells in G0/G1 phase (*p* = 0.023), in comparison with AA monotherapy. Regarding 5-FU monotherapy, CT leads to a significant presence of an apoptotic peak in C2BBe1 cells (*p* = 0.035) and in WiDr cells (*p* = 0.02). Nevertheless, the cell cycle arrest in S-phase in LS1034 cells, observed for 5-FU in monotherapy (*p* < 0.001 relative to control) was not reflected in the CT.

On the other hand, the combination of AA + Oxa induces a significant cell cycle arrest in S-phase for all cell lines (*p* < 0.01). This blockade is significantly higher for CT compared with AA in monotherapy in the case of LS1034 cells (*p* < 0.001), and relatively to Oxa in monotherapy in the case of WiDr cells (*p* < 0.001).

The combination of AA + Iri induced a significant increment in the population of cells in G2/M phase in C2BBe1 (p = 0.011) and in WiDr (*p* = 0.039) cell lines, relatively to AA in monotherapy. In LS1034 cells, CT induced cell cycle arrest in S-phase (*p* = 0.004), that was accomplished by an apoptotic peak (*p* = 0.035), when compared to the treatment with Iri alone.

Thereby, in a general way, the results showed that the combination of AA + Oxa proved to be the most promising CT for all cell lines, being apoptosis the predominant type of induced cell death.

### AA Chemosensitizes CRC Cell Lines to Oxaliplatin

**FIGURE 3:**
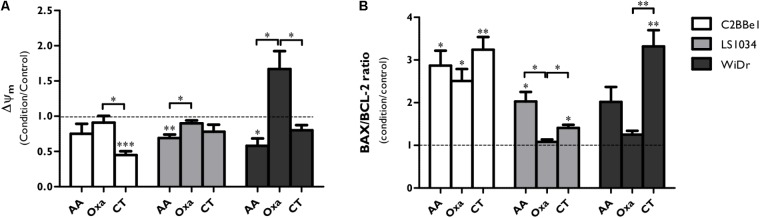
Analysis of BAX and BCL-2 expression and mitochondrial membrane potential (Δψ_m_) after exposure of C2BBe1, LS1034 and WiDr cells to AA and Oxa alone or in CT for 48 h. **(A)** Mitochondrial membrane potential (Δψ_m_) values are expressed as aggregates/monomers (A/M) and **(B)** apoptosis-related proteins are expressed as BAX/BCL-2 ratio for each condition and cell line. The decrease in the A/M ratio is directly correlated with decreased Δψ_m_. All results express the mean and standard deviation of at least four independent experiments, in duplicate. Statistically significant differences are marked with ^∗^*p* < 0.05, ^∗∗^*p* < 0.01, and ^∗∗∗^*p* < 0.001.

Considering that CT of AA + Oxa induces cell death mainly by apoptosis, mitochondrial membrane potential (Δψ_m,_ **Figure 3A**) was evaluated, as well as the expression of apoptosis-related proteins through the ratio between pro BAX/BCL-2 (pro-/anti- apoptotic proteins, **Figure 3B**).

The CT of AA + Oxa induced alterations on the Δψ_m_ (**Figure 3A**). In C2BBe1 cells, CT significantly decreased the Δψ_m_ by half compared to control (*p* < 0.001) and to Oxa alone (*p* < 0.05), while in the other two cell lines just a slight decrease was observed. It should be noted that in WiDr cells, Oxa induced a significant increase of Δψ_m_ compared to AA alone (*p* = 0.014) and to CT (*p* = 0.044).

As can be seen in **Figure 3B**, CT induced a significant increase of BAX/BCL2 ratio in 3.2 times (*p* = 0.006) for C2BBe1 cells, 1.4 times (*p* = 0.011) for LS1034 cells and 3.3 times (*p* = 0.007) for WiDr cells, when compared to control. This ratio was also statistically higher compared to both monotherapies in the case of LS1034 cell line (*p* < 0.05) and compared to Oxa alone in WiDr cell line (*p* = 0.007).

**FIGURE 4:**
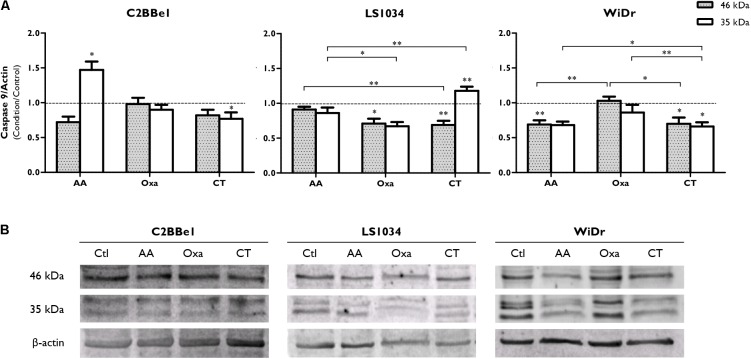
Analysis of caspase-9 expression after exposure of C2BBe1, LS1034 and WiDr cells to AA and Oxa alone or in CT for 48 h. **(A)** The plot represents the expression of caspase-9 (46 kDa) and a 35 kDa cleavage product. The results are expressed as the ratio between the fluorescence intensity of caspase-9 and β-actin, normalized to the control. All results express the mean and standard error of at least six independent experiments. Statistically significant differences are marked with ^∗^*p* < 0.05 and ^∗∗^*p* < 0.01. **(B)** Representative immunoblot of caspase-9 expression.

Caspase-9 expression results, showed in **Figure 4** and Supplementary Figure 1, denoted an activation after AA treatment in C2BBe1 cells, with a tendency to decrease pro-caspase-9 (46 kDa) and a statistically significant increase in its cleaved protein (35 kDa, *p* = 0.041); however, after the use of CT, there was just a significant decrease of the cleaved protein (*p* = 0.046). Concerning LS1034 cells, pro-caspase-9 expression decreased to 0.69 ± 0.06 (*p* = 0.006) followed by an increase of 1.18 times of the cleaved protein (*p* = 0.010), denoting caspase-9 activation. In the other hand, in WiDr cells, CT decreased both caspase-9 (0.66 ± 0.06; *p* < 0.05) and pro-caspase-9 (0.70 ± 0.09; *p* < 0.05) expressions.

**FIGURE 5:**
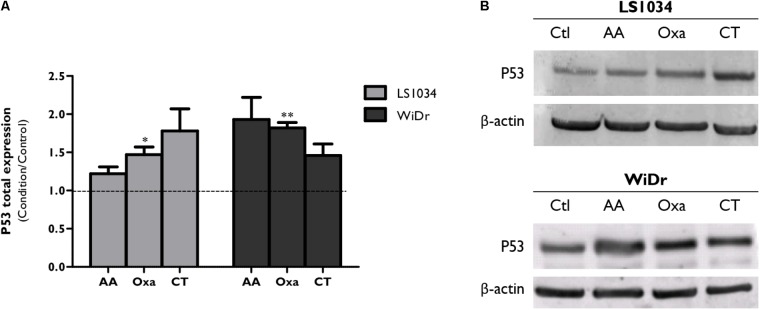
Analysis of P53 expression after exposure of LS1034 and WiDr cells to AA and Oxa alone or in CT for 48 h. **(A)** The plot represents the expression of P53 as the ratio between the fluorescence intensity of P53 and β-actin, normalized to the control. All results express the mean and standard error of at least five independent experiments. Statistically significant differences are marked with ^∗^*p* < 0.05 and ^∗∗^*p* < 0.01. Panel **(B)** is a representative immunoblot of P53 expression.

Considering the role developed by P53 in cell death processes, its expression was evaluated (**Figure 5** and Supplementary Figure 2). Thus, P53 expression increased after monotherapy and CT in LS1034 and WiDr cells, with statistically significant differences only with Oxa treatment. Alterations with CT were also seen, since it increased P53 expression to 1.78 ± 0.29 in LS1034 cells and to 1.46 ± 0.15 in WiDr cells.

### AA Inhibits Tumor Growth and Increases the Efficacy of Irinotecan and Oxaliplatin

**FIGURE 6:**
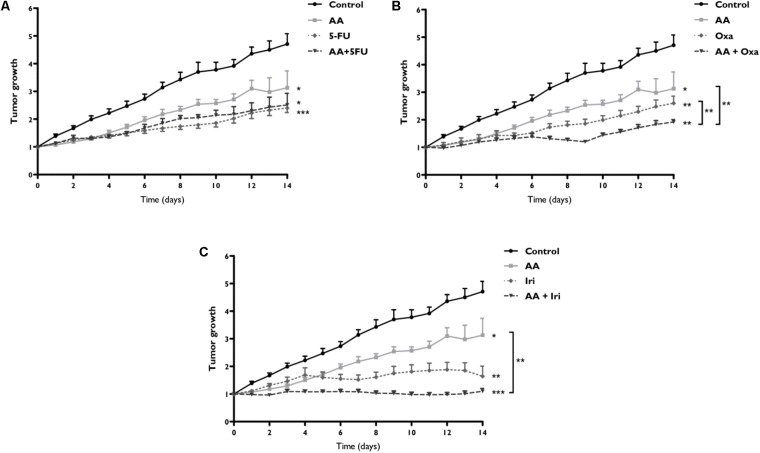
Assessment of tumor growth after therapy with AA and 5-FU **(A)**, Oxa **(B)** or Iri **(C)** alone or in combined therapy, in a WiDr tumor xenograft. Xenotransplanted animals were divided into eight groups: control group, not subjected to any treatment (*n* = 6); AA group, subjected to AA alone (*n* = 8); 5-FU group, subjected to 5-FU alone (*n* = 6); AA + 5-FU group, subjected to combination therapy of AA and 5-FU (*n* = 4); Oxa group, subjected to oxaliplatin alone (*n* = 6); AA + Oxa group, subjected to combination therapy of AA and Oxa (*n* = 6); Iri group, subjected to irinotecan alone (*n* = 6); and AA + Iri group, subjected to combination therapy of AA and Iri (*n* = 8). Tumor growth expresses the tumor volume relative to day 0. Statistically significant differences are marked with ^∗^*p* < 0.05, ^∗∗^*p* < 0.01, and ^∗∗∗^*p* < 0.001.

After the assessment of the effect of AA combined with the three chemotherapeutic drugs *in vitro*, a heterotopic xenograft model of WiDr cell line was used to ascertain the effect of AA on tumor growth and aggressiveness, as well as its efficacy in tumor sensitization to the chemotherapeutic agents conventionally used and previously studied, namely 5-FU, Oxa and Iri (**Figure 6**).

Daily AA doses inhibited tumor growth (*p* = 0.015), presenting after 14 days 1.5 times lower tumor growth rate compared to control group (absence of treatment). CT of AA + 5-FU (**Figure 6A**) only showed benefits relatively to control group (*p* < 0.001). On the other hand, the combination of AA + Oxa, showed in **Figure 6B**, induced an inhibition of tumor growth with statistically significant differences compared to AA (*p* = 0.003) and Oxa (*p* = 0.010) monotherapies. Concerning the combination of AA + Iri (**Figure 6C**) a stagnation in tumor growth was noticed, corresponding the maximum growth rate to 1.10 ± 0.10, which was statistically lower compared with control group (*p* < 0.001) and AA monotherapy group (*p* = 0.002).

**FIGURE 7:**
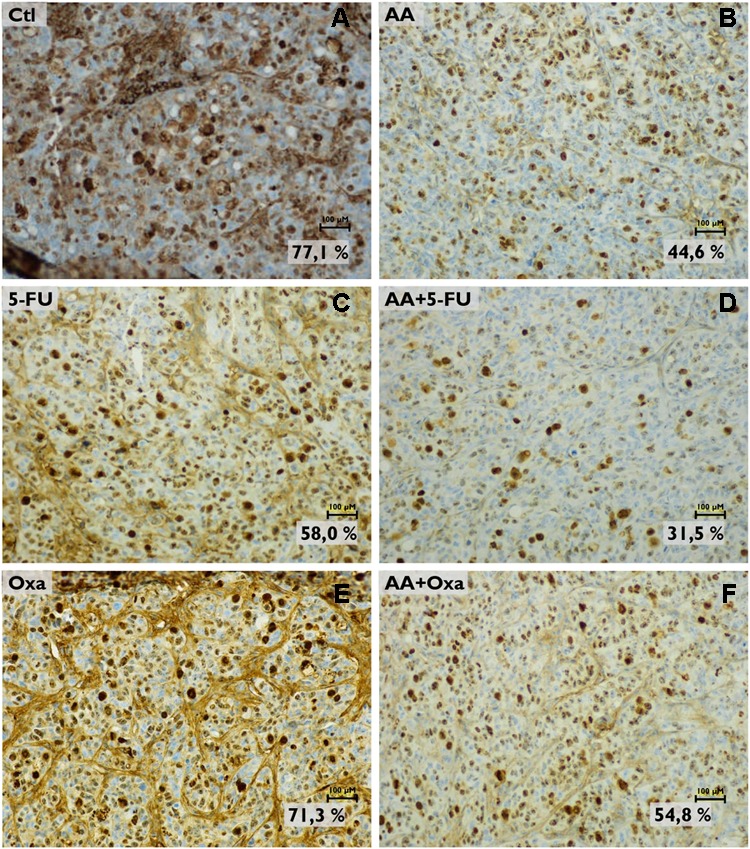
Analysis of KI-67 expression in tumor histological sections by immunohistochemistry. Images are representative of the control **(A)**, the AA treatment **(B)**, the 5-FU treatment **(C)** the AA and 5-FU combined treatment **(D)** the oxaliplatin (Oxa) treatment **(E)** and the AA and Oxa combined treatment **(F)** conditions. Images were obtained with a magnification of 200x (scale bars: 100 μM). Proliferative index is based on KI-67 expression.

These results were confirmed with the determination of proliferative index, which was assessed by KI-67 (a proliferation marker) staining (**Figure 7**). Tumors submitted to AA treatment (**Figure 7B**) showed a decrease of the proliferation index to 44.6%, compared with control group (77.1%; **Figure 7A**). As expected, when compared to control group, a decrease in proliferative index was observed after 5-FU treatment (58.0%; **Figure 7C**), which decreased further in the presence of AA (31.5%; **Figure 7D**). AA also sensitized tumor cells to Oxa, since with Oxa monotherapy (**Figure 7E**) the proliferative index was 71.3% and when in combination with AA (**Figure 7F**) this value decreased to 54.8%.

## Discussion

Previous evidences on the cytotoxic effect of AA (Chen et al., 2005; Kawada et al., 2013; Mastrangelo et al., 2015; Gillberg et al., 2017), prompt its inclusion in a combination regimen with drugs commonly used in conventional chemotherapy, namely for the treatment of CRC (Pires et al., 2016).

Vitamin C has been studied *in vitro* and *in vivo* in combination with numerous cytotoxic drugs (Wilson et al., 2014). Although the controversial opinions regarding its efficacy when combined with conventional radiotherapy or chemotherapy, several *in vitro* studies suggest that ascorbate, at pharmacological concentrations, may increase the efficacy of these treatments (Du et al., 2012). The chemosensitizing effect of vitamin C has already been proven by several authors in various types of cancer (Kurbacher et al., 1996; Verrax and Calderon, 2009; Espey et al., 2011; Vollbracht et al., 2011; Ma et al., 2014; Lee et al., 2017). Thus, as already shown, ascorbate given in intravenous pharmacological concentrations, may not only potentiate the effects of conventional chemotherapy, but also improve the quality of life of cancer patients (Du et al., 2012).

Considering that reduced sensitivity of some tumors to chemotherapy and the highly associated adverse effects continue to be some of the major obstacles in the effective treatment of CRC (Gonzalez-Pons and Cruz-Correa, 2015), this paper aimed to study the potential of a new therapeutic approach, effective against this neoplasia with diminished side effects for the patient. This approach was based on the study of the combination of high concentrations of AA with reduced concentrations of drugs conventionally used in CRC patients and eligible for first and second line chemotherapy regimens. The evaluation of the potential synergy between AA and 5-FU, Oxa or Iri was first made using *in vitro* studies and later *in vivo* studies.

In general, high concentrations of AA sensitized the three CRC cell lines to the effect of 5-FU, Oxa and Iri. The most notorious anti-proliferative effects were observed when AA was present in greater proportion.

High concentrations of AA sensitized the three cell lines under study to the effect of 5-FU. Although the presence of AA induced a decrease in the IC_50_ of 5-FU at 48 h, the two compounds just acted synergistically for longer incubation times. Previous studies also reported that AA reinforced the anti-proliferative activity of 5-FU (Abdel-Latif et al., 2005; Verrax and Calderon, 2009), however, our results show that, at 48 h, this anti-proliferative synergy is not translated in terms of cytotoxicity, since no significant differences are seen between CT and AA alone.

The effects of AA combined with Iri were synergistic for LS1034 cells, inducing cell cycle dysregulation, with emphasis on S-phase arrest, and increasing cell death populations. Combined treatment induced a reduction of 11.5% and 43% in cell viability compared with AA or Iri therapies, respectively, emphasizing the synergistic effect previously demonstrated. The lack of synergy in C2BBe1 and WiDr cell lines highlight a possible mechanism of synergy that can be influenced by intrinsic characteristics of the cell lines. Iri is converted into its active metabolite, SN-38, by carboxylesterases, enzymes with high levels of liver activity, but also in the duodenum, jejunum, ileum, colon and rectum (Rothenberg, 2001; Encarnação et al., 2018). Thus, both levels of carboxylesterases intrinsic to each cell line and the limitation of the *in vitro* experimental model associated with the absence of these enzymes from normal human tissues may influence the response to Iri therapy and consequently to combination therapy with AA. In this way, *in vivo* studies are expected to more accurately reflect the synergistic potential of this combination. Even so, the results obtained for LS1034 cells are quite promising considering the multi-drug resistance of this cell line associated with P-glycoprotein (PGP) overexpression (Casalta-Lopes et al., 2011). In fact, both Iri and SN-38 are substrates of this efflux protein (Jonsson et al., 2000; Sooryakumar et al., 2011), so its extrusion of the cell by PGP would be expected to increase resistance to treatment. However, not only a cytotoxic effect occurred with treatment with Iri alone, but also this effect was further potentiated by the presence of AA.

The association of AA with Oxa showed very promising results, considering that a synergistic effect was demonstrated, in almost all conditions and in the three cell lines under study. Although the use of lower AA concentrations could bring better insights on the potential of the association of AA and chemotherapeutic drugs, in general, CT induced cell cycle arrest in S-phase, along with a statistically significant decrease on cell viability, with respect to Oxa and, in some cases, to AA. Nevertheless, the synergistic anti-cancer mechanisms of CT differ according to the cell line.

In C2BBe1 cells, AA and Oxa seem to act synergistically by the activation of the intrinsic pathway of apoptosis, translated on the statistically significant increase of the ratio between BAX and BCL-2 proteins, which in turn is associated with a decrease of Δψ_m_. In AA treatment, the activation of caspase-9 is evident, while the combined treatment induced a slight decrease in the caspase-9 cleaved protein, which may be related to the presence of necrosis (Golstein and Kroemer, 2007). Previous results obtained by our group showed that AA mediates reactive oxygen species (ROS) formation capable of irreparably damaging DNA (Pires et al., 2016). Golstein and Kroemer (2007) have also proven that Oxa induces DNA damages via both P53-dependent and -independent pathways. The null-P53 expression of C2BBe1 cells may explain the increased susceptibility of this cell line to DNA damage promoted synergistically by AA and by Oxa, given the inherent failure to regulate cellular processes, such as cell cycle control, DNA repair and transcription. The oxidative role of AA may be a key factor on the synergistic anti-cancer mechanism, reason why it should be further explored.

In LS1034 cell line, we verified the existence of a complementarity of AA and Oxa cytotoxic mechanisms. AA is described to activate the intrinsic pathway of apoptosis in this cell line (Pires et al., 2016), while Oxa is capable of activating the extrinsic pathway (Zanardelli et al., 2015). The combined treatment culminated in apoptosis, with an increase of BAX/BCL-2 ratio, a tendency to a decrease of Δψ_m_ and activation of caspase-9. The intrinsic and extrinsic pathways of apoptosis converge on effector caspases, namely caspase-3, -6 and -7. The production of bH3 interacting-domain death agonist (tBID) by caspase-8 in the extrinsic pathway will increase the action of BAX and BAK proteins, by enhancing the intrinsic pathway and magnifying the apoptotic response (Baig et al., 2016). AA and oxaliplatin, acting through different anti-cancer mechanisms constitute an advantage in this combination regimen, becoming able to overcome the characteristic multi-drug resistance of these cells.

In WiDr cells, cell viability results evidenced the predominant increase in cell death by late apoptosis/necrosis, in response to the combined treatment. Interestingly, Oxa alone induced an increase of Δψ_m_ concomitant with mitochondrial hyperpolarization (Mamede et al., 2015). Several authors argue that mitochondrial hyperpolarization occurs transiently and reversibly prior to the activation of apoptosis (Perl et al., 2004; Nagy et al., 2007). In more detail, its occurrence was demonstrated to happen before activation of caspases, phosphatidylserine externalization and Δψ_m_ disruption in apoptosis, via the death receptor pathway (Banki et al., 1999) or by the action of H_2_O_2_ (Puskas et al., 2000). Mitochondrial hyperpolarization thus precedes dilatation and rupture of the outer mitochondrial membrane and the release of apoptogenic factors into the cytoplasm (Ly et al., 2003). Thus, Oxa alone did not induce cell death, coexisting with the absence of changes in the BAX/BCL-2 ratio and the expression of caspase-9. However, an increased stimulus with the presence of AA was enough for the apoptotic and/or necrotic process to be completed. CT showed then an increase in the BAX/BCL-2 ratio and inhibition of caspase-9, indicating a possible predominance of apoptotic cell death. Moreover, P53 is known to be mutated in about 50% of CRC and that the mutated state of this tumor suppressor protein is implicated in the increase or decrease of tumor sensitivity to numerous chemotherapeutic drugs (Golstein and Kroemer, 2007; Pires et al., 2016). In fact, in WiDr cells expressing mutant P53, AA and Oxa also induced a slight increase of P53 expression levels, proving that this combination also acts synergistically in P53-mutated tumors.

*In vivo* studies have been an essential complement to *in vitro* studies, with a few key facts highlighted. The combination of 5-FU with AA showed no benefit compared to 5-FU alone. In contrast to the lack of synergy seen in *in vitro* studies with the combination of AA with Iri, the animal model revealed the therapeutic potential of this combination. The combination of AA with Oxa caused a stagnation of the tumor growth rate, again being the most promising tested combination.

## Conclusion

Our work demonstrated the increased efficacy of Iri and Oxa against CRC, by its association with pharmacological AA concentrations. These pre-clinical studies also revealed the potential of a combined regimen based on AA and conventional chemotherapeutic drugs in cell lines with more aggressive phenotypes, namely, tumors with mutant or null P53 expression and tumors resistant to chemotherapy. This work highlights the need for the prosecution of randomized and double-blind clinical trials with CRC patients, whose treatment includes drug combination regimens with Oxa and Iri with pharmacological vitamin C concentrations.

## Author Contributions

AP, AS-R, AA, and MB contributed to the study conception, design and follow-up. AP, CM, and JE performed the *in vitro* and *in vivo* studies, except for some specific methodologies. ML performed the western blot studies. AG analyzed flow cytometry data. RO performed the histological analysis. AP and JC-L performed the statistical analysis. AP was responsible for data analysis and interpretation. AP, CM, and IM wrote sections of the manuscript. AP, CM, and MB read and critically reviewed the submitted version of the manuscript. All authors provided approval for publication of the content.

## Conflict of Interest Statement

The authors declare that the research was conducted in the absence of any commercial or financial relationships that could be construed as a potential conflict of interest.
