# Supplementary material for: Ascorbic Acid Chemosensitizes Colorectal Cancer Cells and Synergistically Inhibits Tumor Growth
Source: Front Physiol. 2018 Jul 23;9:911. doi: 10.3389/fphys.2018.00911 (PMC6064950; doi:10.3389/fphys.2018.00911)
Supplement: Supplementary file 1 [file Image_1.PDF]

Supplementary Material

Ascorbic acid chemosensitizes colorectal cancer cells and synergistically inhibits tumor growth

Pires AS[1,2,3]*, Marques CR[1,2], Encarnação JC[1,3], Abrantes AM[1,3], Marques IA[1,3], Laranjo M[1,3], Oliveira R[1,3,4], Casalta-Lopes JE[1,3], Gonçalves AC[3,5], Sarmento-Ribeiro AB[3,5,6], Botelho MF[1,3]

*** Correspondence:** Corresponding Author: a.salome.pires@gmail.com

1 Supplementary Images

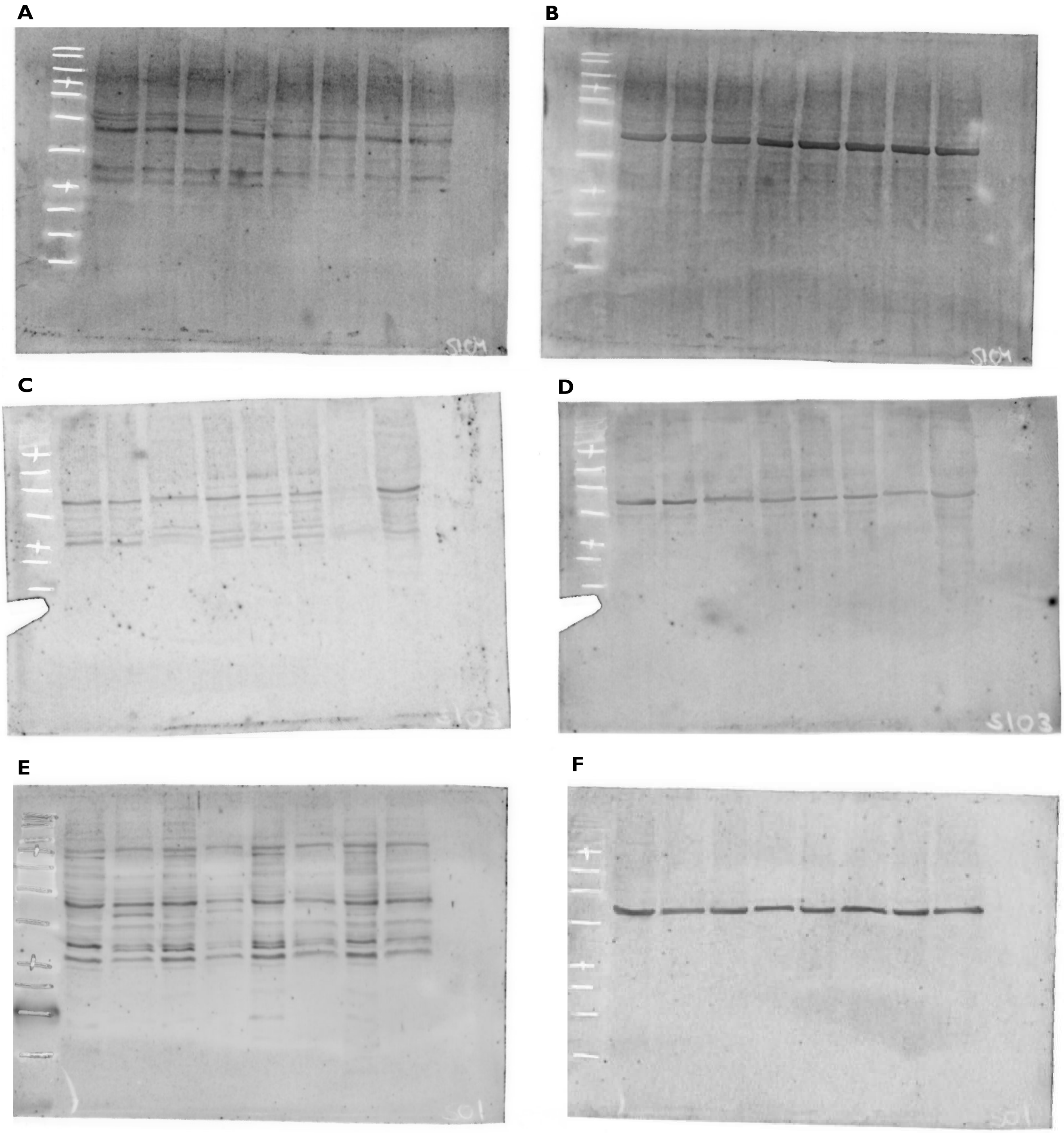

Supplementary Figure 1 – Uncropped images of analysis of caspase-9 expression after exposure of C2BBe1 (A and B), LS1034 (C and D) and WiDr (E and F) cells to AA and oxaliplatin (Oxa) alone or in combined therapy for 48 hours. Panels A, C and E are the immunoblots of caspase-9 (46 kDa) and a 35 kDa cleavage product. Panels B, D and F are the immunoblots of β-actin, used as protein content control. Although some unspecific bands were obtained, the bands were quantified based on the molecular weight of interest.
