# Supplementary material for: Ascorbic Acid Chemosensitizes Colorectal Cancer Cells and Synergistically Inhibits Tumor Growth
Source: Front Physiol. 2018 Jul 23;9:911. doi: 10.3389/fphys.2018.00911 (PMC6064950; doi:10.3389/fphys.2018.00911)
Supplement: Supplementary file 2 [file Image_2.PDF]

Supplementary Material

Ascorbic acid chemosensitizes colorectal cancer cells and synergistically inhibits tumor growth

Pires AS[1,2,3]*, Marques CR[1,2], Encarnação JC[1,3], Abrantes AM[1,3], Marques IA[1,3], Laranjo M[1,3], Oliveira R[1,3,4], Casalta-Lopes JE[1,3], Gonçalves AC[3,5], Sarmento-Ribeiro AB[3,5,6], Botelho MF[1,3]

*** Correspondence:** Corresponding Author: a.salome.pires@gmail.com

1 Supplementary Images

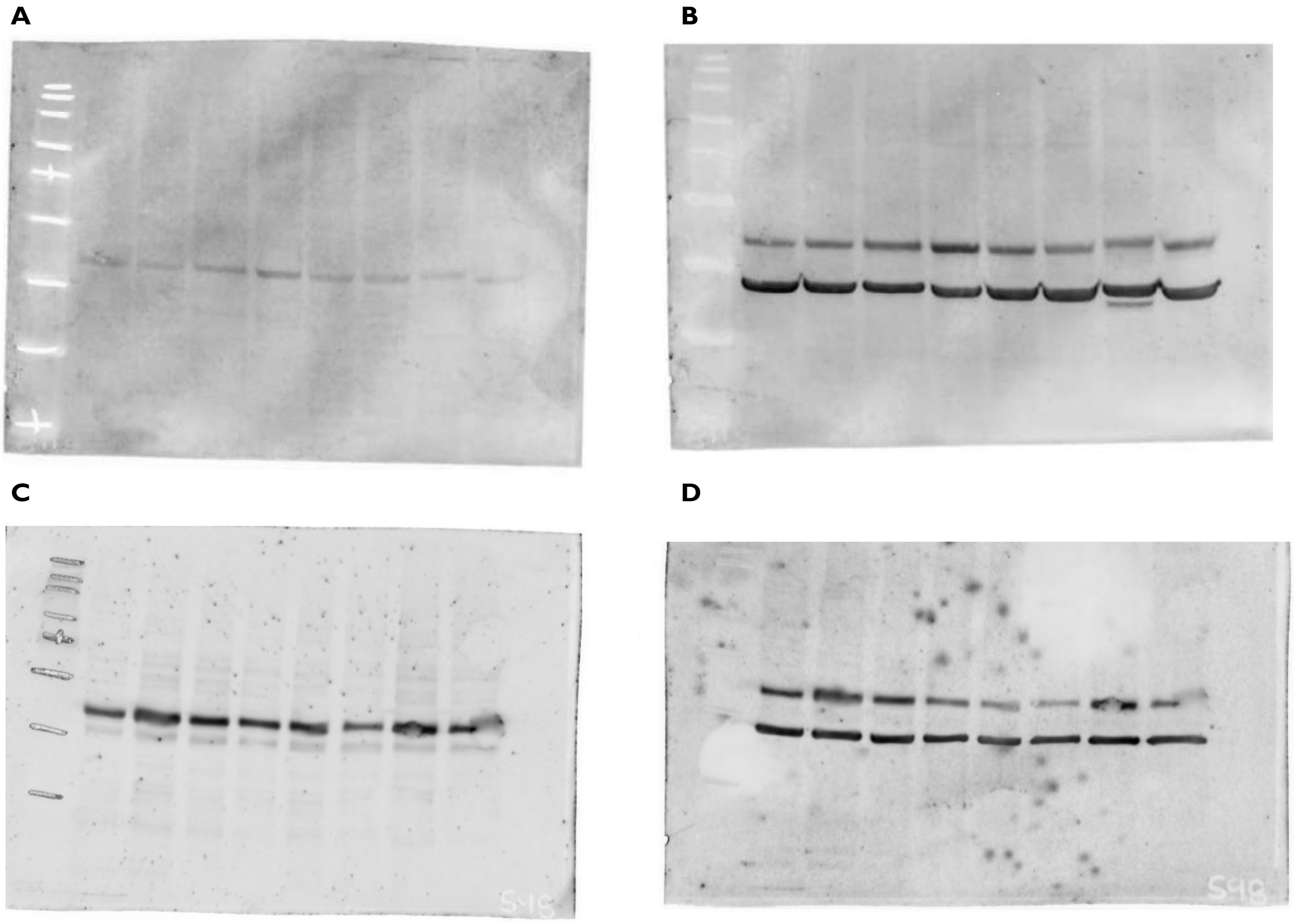

Supplementary Figure 2 – Uncropped images of analysis of P53 expression after exposure of LS1034 (A and B) and WiDr (C and D) cells to AA and oxaliplatin (Oxa) alone or in combined therapy for 48 hours. Panels A and C are the immunoblots of P53. Panels B and D are the immunoblots of β-actin, used as protein content control.
